# Scaling photonic lanterns for space-division multiplexing

**DOI:** 10.1038/s41598-018-27072-2

**Published:** 2018-06-11

**Authors:** Amado M. Velázquez-Benítez, J. Enrique Antonio-López, Juan C. Alvarado-Zacarías, Nicolas K. Fontaine, Roland Ryf, Haoshuo Chen, Juan Hernández-Cordero, Pierre Sillard, Chigo Okonkwo, Sergio G. Leon-Saval, Rodrigo Amezcua-Correa

**Affiliations:** 1 CREOL, The College of Optics & Photonics, the University of Central Florida, Orlando, Florida 32816-2700 USA; 2 Bell Laboratories/Alcatel-Lucent, 791 Holmdel Rd., Holmdel, New Jersey 07733 USA; 3 Instituto de Investigaciones en Materiales, UNAM, Cd Universitaria, Ciudad de México, 04510 Mexico; 4 Prysmian Group, Parc des Industries Artois Flandres, 644 boulevard Est, Billy Berclau, 62092 Haisnes Cedex, France; 5 Institute for Photonic Integration, Flux, Groene Loper 5, 5612 AE Eindhoven, The Netherlands; 6 Institute of Photonics and Optical Science, School of Physics, The University of Sydney, New South Wales, 2006 Australia; 7 Present Address: Instituto de Ciencias Aplicadas y Tecnología, UNAM, Cd. Universitaria, Mexico City, 04510 Mexico

## Abstract

We present a new technique allowing the fabrication of large modal count photonic lanterns for space-division multiplexing applications. We demonstrate mode-selective photonic lanterns supporting 10 and 15 spatial channels by using graded-index fibres and microstructured templates. These templates are a versatile approach to position the graded-index fibres in the required geometry for efficient mode sampling and conversion. Thus, providing an effective scalable method for large number of spatial modes in a repeatable manner. Further, we demonstrate the efficiency and functionality of our photonic lanterns for optical communications. Our results show low insertion and mode dependent losses, as well as enhanced mode selectivity when spliced to few mode transmission fibres. These photonic lantern mode multiplexers are an enabling technology for future ultra-high capacity optical transmission systems.

## Introduction

The swift growth in optical communications in recent years requires an increase in data transmission capabilities thereby entailing a growth in the capacity of optical networks. Current single-mode fibre (SMF) systems are rapidly approaching the theoretical non-linear Shannon limit, and most of the available degrees of freedom for scaling their capacity have been already exploited^1^. Recently, space-division multiplexing (SDM) has been envisioned as an alternative to exploit different spatial modes as independent transmission channels to increase data transmission capacity through a single optical fibre^2^. Historically, multimode fibres (MMFs) were limited to short distance links due to mode mixing and the need for complex signal processing to undo the mode scrambling. The advent of few-mode fibres (FMFs), capable to transmit solely a few spatial modes with limited crosstalk, has attracted a new interest in multimode (MM) systems^3^. Likewise, the use of a few-mode multicore fibre has been also demonstrated as an alternative to increase the transmission capacity^4^. In addition to SDM, communication systems based on FMFs offer the potential to increase the transmission capacity in optical fibre links and develop long-haul links using the same number of components typically used in SMF systems^5–7^. Nevertheless, critical components to effectively connect single-mode to multimode devices are still needed.

Photonic lanterns (PLs) are spatial mode converters connecting single-mode signals from multiple individual waveguide cores into a single multimode waveguide, fabricated either by using optical fibres or planar waveguides^8^. These are key component in SDM systems for converting single-mode signals into different modes supported by a multimode waveguide and vice versa^8,9^. Specific spatial modes can be generated using free-space optical elements and adaptive optics, e.g. phase plates^10^. Usually, these methods result in complex setups based on multiple optical elements thereby involving alignment difficulties and generally yielding high loss, unstable signals, and limited coupling efficiency to optical fibres. Although methods based on diffractive elements and integrated photonics have shown to yield compact and integrated devices for spatial mode generation^11,12^, waveguide-based devices are always preferred owing to their natural compatibility with optical fibres.

All-fibre PLs were foremost developed for astronomy applications^8^, and more recently, they have been implemented as multiplexer/demultiplexer (MUX/DEMUX) devices in optical communications for SDM applications^7,9,13,14^. Furthermore, they have also proven useful for developing other photonic devices such as sensors and multi-Watt level mode-selective amplifiers^15,16^. Since PLs can selectively generate different mode profiles, ongoing research aims at increasing the capabilities to generate even higher order of spatial modes. Mode selectivity in PLs refers to the capability of the device for accessing each of the available spatial modes, and play a fundamental role in SDM systems because it allows for compensating mode dependent effects, such as differential group delay (DGD) and mode dependent losses (MDL)^9,17^. The demand of these devices entails the fabrication of PLs with a larger number of fibres, each of them intended to excite a specific mode and with reduced mode crosstalk.

In this paper, we demonstrate a novel approach for scaling the fabrication of all-fibre mode-selective photonic lanterns with a higher number of modes and the capability to achieve modal selectivity. The procedure is based on the use of special microstructured performs, specifically designed to contain and hold the fibres in the particular geometrical arrangement required to generate up to 15 spatial modes. Upon selecting the proper fibre core diameters and positioning them in the adequate location within the preform, we were able to fabricate mode-selective PLs yielding up to 9 different pure LP fibre modes. Furthermore, PLs were designed to directly splice them to transmission fibres, thus providing full integration with all the single-mode based photonic technology for communications. While the mode generation capabilities were evaluated by direct observation of the output light patterns, mode selectivity was analysed upon splicing the PLs to FMFs and obtaining the transfer matrices. The proposed fabrication method is shown to be useful for obtaining PLs that can be scaled up to a higher number of modes and conveniently spliced to FMFs for launching higher order fibre modes at low-loss and low cross-talk manner.

## Photonic Lantern Fabrication

Fabrication of all-fibre photonic lanterns involves deploying a circular array of multiple fibres subsequently fused and stretched until a multicore fibre array is created. In general, the fibres used are single-mode and these are placed inside a glass capillary tube with lower refractive index than that of the fibres cladding material; subsequently, the capillary is adiabatically tapered down to the fibre size. After stretching, the SMFs collapse all together resulting in a MMF structure, with the fused fibres acting as the new core and the low-index capillary as its cladding^8,9^. In principle, the number of spatial modes supported by the tapered end of the PLs is equal to the number of fibres used in the device^8^.

**Figure 1:**
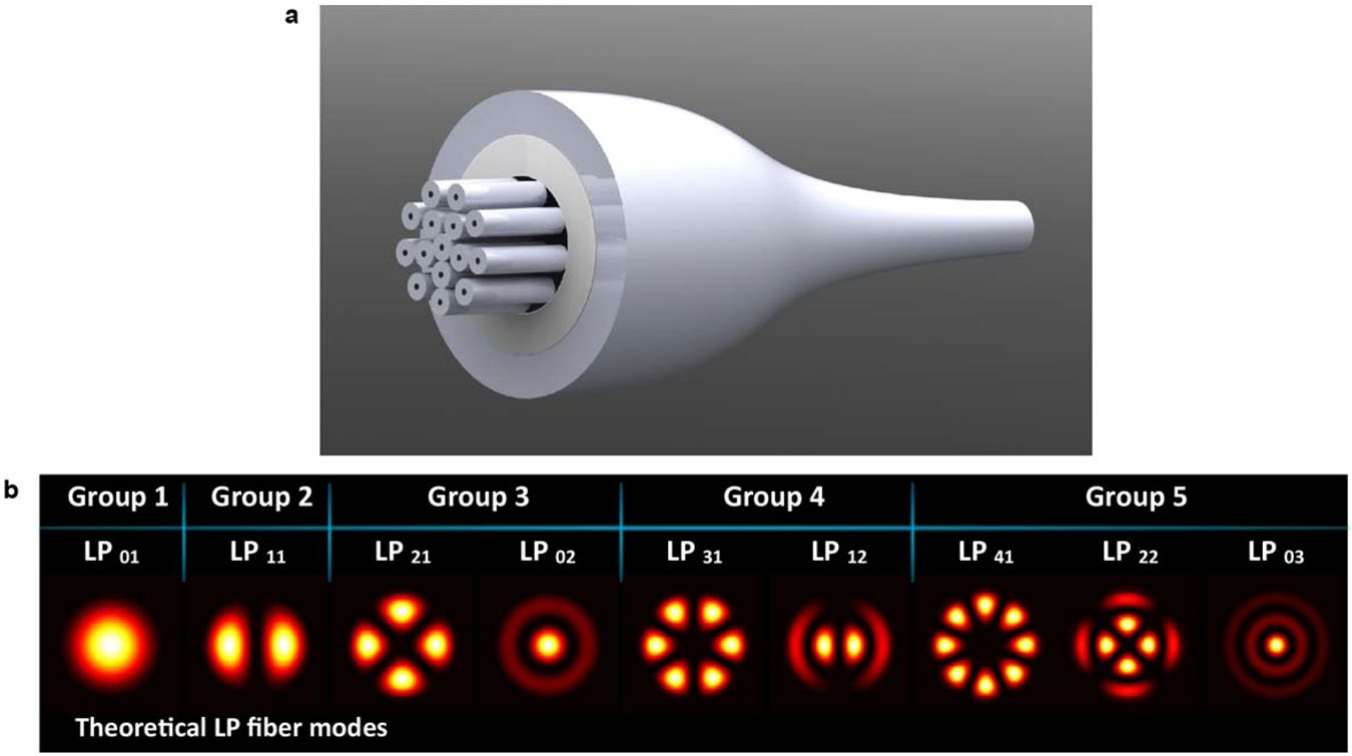
All-fibre photonic lantern. (**a**) Illustration of a photonic lantern fabricated using 15 fibres, and (**b**) theoretical LP fibre modes attainable with this device.

Upon launching light into the SMFs, coupling among the different fibre cores along the PL transition length occurs generating different mode profiles at the MMF section. The intensity distribution in the resulting mode profiles strongly depends on the coupling conditions within the transition section of the structure, which depends on the propagation features of the fibres^18^. Similarly, the spectral features of the PLs is defined by the number and types of fibres, their position within the capillary, the refractive index difference between the fibre cladding and capillary, and the tapering ratio^8,19,20^. In order to integrate the PLs into full transmission systems, the size and numerical aperture (NA) of the raised MMF must be adequate to match those of the FMF intended for the MM transmission system. Figure 1 depicts an all-fibre PL together with the 9 LP modes that could be generated using 15 fibres with appropriate sizes and correct locations within the capillary tube.

Mode-selective photonic lanterns (MSPLs) allow for the selective excitation of diverse LP modes launching single-mode light signals individually into each of the fibres that conform the device^19–22^. This effect is achieved by breaking the degeneracy between the modes of the uncoupled fibre cores, thus ensuring different propagation constants for each supported mode in the final MMF^19,20^. An approach to fulfil these conditions involves using optical fibres with different core diameters, thus yielding different coupling conditions among all the fibres along the transition length in the PL. Because mode scrambling inevitably occurs during transmission through FMFs, full modal selectivity may not be imperative for SDM applications. Hence, instead of addressing individual LP modes, modal selectivity can be done by mode groups. This is achieved with mode-group-selective PLs (MGS-PLs), in which each fibre can excite an orthogonal combination of the modes contained within a mode group^22^. These devices are suitable for SDM purposes, since multiple-input multiple-output (MIMO) processing can be readily used for mode unscrambling^7,9^. All-fibre MSPLs with different fibre core diameters have been successfully fabricated for two, four, and six LP modes with negligible mode mixing^19–21,23,24^. Likewise, MGS-PLs with three and six fibres have been fabricated and tested for SDM applications^22^. Regarding PLs with a larger number of fibres, 15-fibre devices but without modal selectivity have been fabricated and tested in SDM transmission experiments^14,23^.

## Microstructured Preforms for Photonic Lantern Fabrication

**Figure 2:**
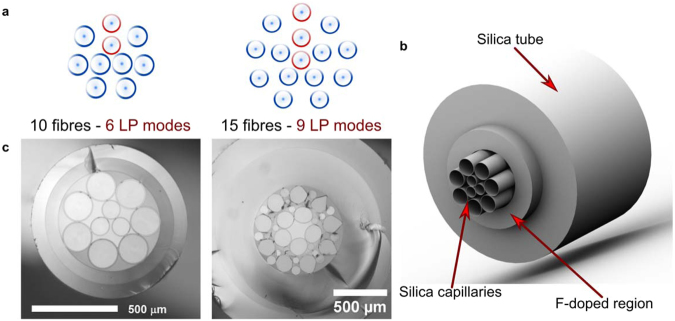
SMFs distribution for higher-order all-fibre photonic lanterns. (**a**) Spot array configurations required for the 10- and 15-fibre PLs construction^17^. (**b**) Illustration of a microstructured preform for PLs fabrication, and (**c**) microscope images of the microstructured preforms assembled for PLs fabrication.

Scaling the PLs to support higher order modes involves a larger number of single-mode fibres. The precise spatial distribution of independent light spots required to excite the modes supported by a given FMF can be estimated through coupling matrix calculations^17,18^. These dictate the necessary arrangement of fibre cores for PLs considering the LP_*lm*_ fibre modes required to excite in the FMF^17^. As an example, the spots distributions required for PLs with 10 and 15 single-mode fibres are depicted in Fig. 2a. As seen in the figure, a ring of fibres is required in the fibre array for each value of the radial index *m*. Each ring is formed with a number of fibres given by 2*l*_*Max*,*m*_ + 1, where *l*_*Max*,*m*_ is the largest value of the azimuthal index (*l*), for each value of *m*^9,17^. Hence, the positions for the fibres include one spot for every non-degenerate mode and two spots per degenerate mode. Although the fibre distribution can be readily determined, the specific fibre arrangements cannot be obtained by conventional fibre stacking. Hence, fibre positioning for PLs supporting higher order modes becomes a particularly challenging task.

In order to circumvent the problems for allocating the fibres within the capillary, we used microstructured preforms allowing holding 10 and 15 fibres in the desired positions^9,23,25^. The preforms were fabricated upon stacking synthetic silica capillaries inside a fluorine-doped tube, as illustrated in Fig. 2b. This structure was subsequently drawn to obtain the desired size for feeding the single-mode fibres into each capillary. Thin-wall synthetic silica glass capillaries (ID/OD > 0.95) were used to minimize the effects of adding extra silica layers around the fibres. This further helps to maintain the distances between neighbouring SMF cores.

The 10-fibres preform structure contained a central array of three capillaries (ID = 110 μm) stacked in a triangular geometry, surrounded by a ring of seven capillaries (ID = 170 μm). The structure was contained within a low-index fluorine-doped capillary with Δ*n* ≈ −15 × 10^−3^. For the 15-fibres preform, the structure was formed by two concentric ring arrays of nine and five capillaries (ID = 145 μm), respectively, located inside a low-index capillary with Δ*n* ≈ −3 × 10^−3^. Additional silica rods were included in this preform to fill the extra air gaps, and the central region was left unobstructed in order to hold the fifteenth fibre. The resulting preforms had outer diameters of approximately 900 μm and 2 mm for the 10- and 15-fibres preforms, respectively. The refractive index difference of the fluorine-doped capillaries yielded NAs of 0.207 for the 10-fibre, and 0.093 for the 15-fibre arrangements. Optical microscope pictures of the microstructured preforms fabricated in this manner are shown in Fig. 2c. Once these preforms are drawn to the desired size, the required fibres can be threaded into each capillary.

## Photonic Lantern Architecture and Structure

Fabrication of MSPLs using 10 and 15 fibres was successfully achieved upon selection of the appropriate SMF core diameters, their position within the capillary, and suitable tapering parameters. Modal selectivity constrained the use of different core diameters for each of the supported LP modes^9,19^. Due to LP mode degeneracy (with exception of the LP_0*m*_), the construction of the MSPLs required two identical fibres for the excitation of the two corresponding degenerate modes. These fibres with identical core diameters were placed in symmetric positions across the vertical symmetry axis of the arrangement, whereas the fibres with different diameters (intended to excite the LP_0*m*_ modes) were positioned along the vertical axis of the preform (outlined in red in Fig. 2a). In order to minimize mode mixing, the fibres used in the preform were selected to have at least a difference of 2 μm in core diameter for each LP mode. Additionally, graded-index fibres (GIFs, Δ*n* = 16 × 10^−3^) were used because these present low coupling of the fundamental mode to higher order modes over short propagation distances^21,22^. Furthermore, the use GIFs allowed for shorter transition sections for the tapered structure, yielding short and low-loss devices. All the optical fibres used for the devices were fabricated in house.

For the 10-fibres preform, fibres with two different cladding diameters were used: 125 μm and 83 μm. While the fibres with larger diameter were inserted into capillaries forming the outer ring inside the preform, the fibres with smaller diameter were inserted in the three central capillaries. In contrast, the 15-fibres PLs required only fibres with 125 μm cladding diameter. After the assembly process, the fibres/preforms structures were cleaned and placed inside a CO_2_ laser glass processing station (LZM-100, AFL Fujikura). The structures were tapered down and fused together to the proper size for an adequate LP mode generation.

**Figure 3:**
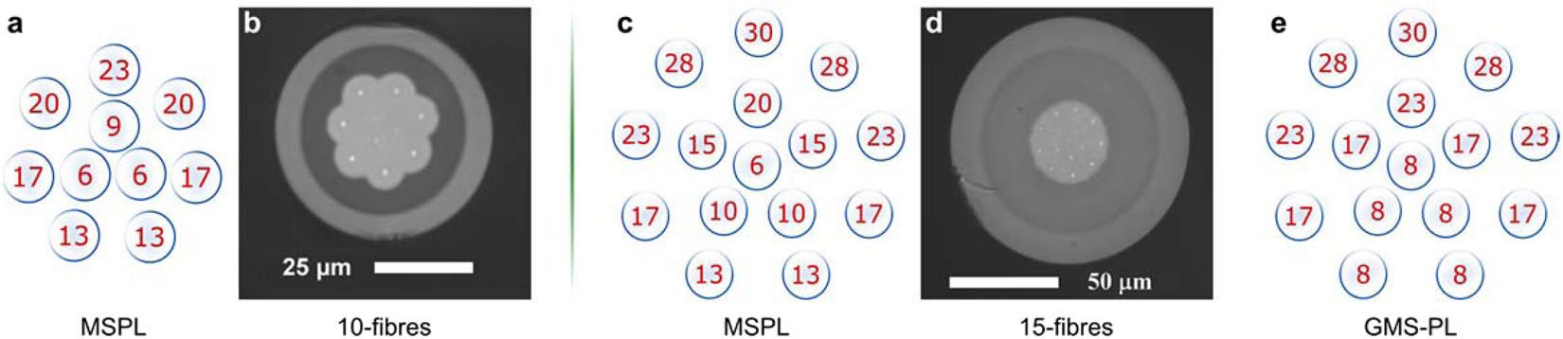
Optical fibre core diameters and positions used for the construction of the PLs. Mode-selective PLs consisting of (**a**) 10 and (**c**) 15 fibres, and the 15-fibres MGS-PL (**e**). Microscope image from the end facet of the cleaved MSPLs: (**b**) 10- and (**d**) 15-fibres.

The MSPL fabricated using ten fibres resulted in a device capable of producing the LP_01_, LP_11_, LP_21_, LP_02_, LP_31_, and LP_12_ modes. In order to generate these modes and to obtain modal selectivity, the fibre core diameters used in the device were 23, 20, 17, 9, 13, and 6 μm, respectively for each of the previously listed LP modes. The processing parameters used to obtain the 10-fibres PLs involved a tapering ratio of 16 with a transition section of 5.75 cm. After the tapering process, we obtained a device supporting the 6 LP modes, with a core size of 27 μm and an outer diameter of 50 μm. Similarly, for the 15-fibres PLs processing was done with a tapering ratio of 20 and transition lengths of 6 cm, yielding a structure with a 35 μm core size and an outer diameter of 105 μm. The resulting 15-fibres MSPLs allowed us to generate the LP_01_, LP_11_, LP_21_, LP_02_, LP_31_, LP_12_, LP_41_, LP_22_, and LP_03_, which were excited with fibre core diameters of 30, 28, 23, 20, 17, 15, 13, 10, and 6 μm, respectively. Figure 3a and c depict the symmetrical distribution and the positions of the fibres with their respective core diameters for both, the 10- and 15-fibres MSPLs. Access to the end facets of the resulting MM structures was achieved through cleaving at the waist section of the tapered devices; microscope images of the cleaved end facets are shown in Fig. 3b and d.

Mode-group-selective PLs were also fabricated using the microstructured preform approach; these devices also serve as mode-multiplexers for SDM applications^22^. In these PLs, the LP modes are classified in groups and remain isolated from the other propagated modes, although mixing can occur among modes within the same group. A photonic lantern addressing LP modes through modal-group selectivity was obtained using the preform designed for holding 15 fibres. Processing of this structure was done preserving the tapering parameters used for the 15-fibres MSPL. The MGS-PL was constructed using five different fibre core diameters, one per mode group: 30, 28, 23, 17, and 8 μm, corresponding to the mode groups 1, 2, 3, 4, and 5. The fibre core distribution for this kind of devices is shown in Fig. 3e.

## PLs Characterization and Spectral Response

**Figure 4:**
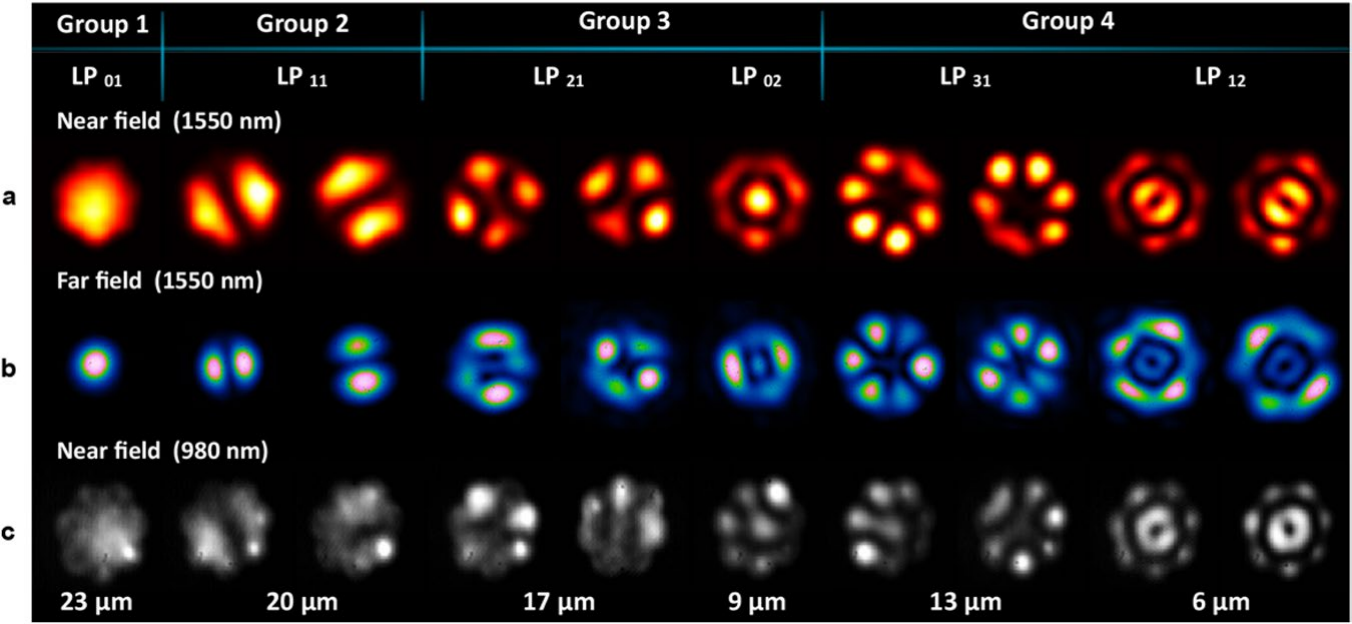
Intensity patterns obtained with the 10-fibers mode selective photonic. (**a**) Near field and (**b**) far field mode profiles at λ = 1550 nm; notice that the degenerate modes (LP_11_, LP _21_, LP_31_, LP_12_) can be independently excited by launching light in the corresponding input fibre. (**c**) Near field mode profiles from the same devices obtained with a laser diode with λ = 980 nm.

**Figure 5:**
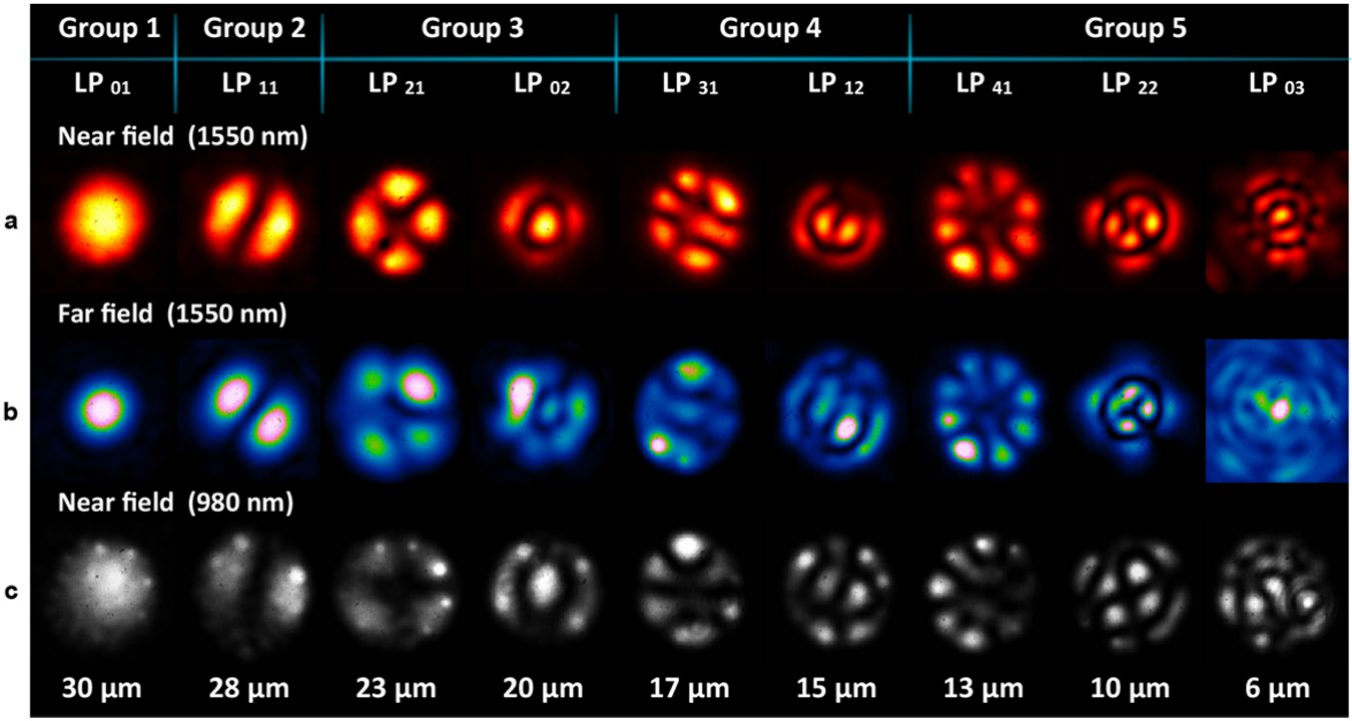
Intensity patterns obtained with the 15-fibres mode selective photonic. (**a**) Near field and (**b**) far field mode profiles at λ = 1550 nm. (**c**) Near field mode profiles from the same devices obtained with a laser diode with λ = 980 nm.

**Figure 6:**
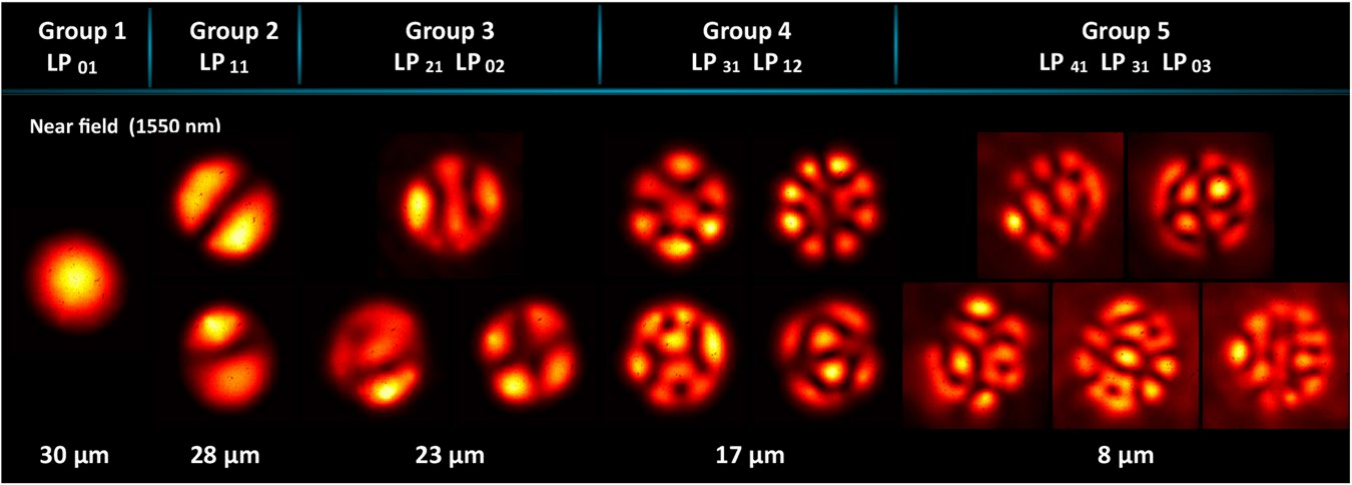
Near field intensity-profiles at λ = 1550 nm from a mode-group-selective photonic lantern fabricated using 15 fibres.

The modal patterns at the output of the fabricated devices were analysed by observing the near field and far field intensity-mode profiles. A broadband light source (50 nm spectral width) from a superluminiscent diode, centred at a wavelength of 1550 nm, was coupled into each of the input fibres. Observation of the near field intensity-mode profiles was performed using a 50X microscope objective and an IR camera. For a thorough characterization, far field intensity-mode profiles were observed for both MSPLs upon removing the microscope objective. The wavelength dependence was also evaluated by launching light from a 980 nm laser diode through the input fibres of the MSPLs. The output intensity-mode profiles from the 10- and 15-fibres MSPLs are shown in Figs 4 and 5, respectively. For the MGS-PL, the near field intensity-mode profiles were only observed at λ = 1550 nm; these are shown in Fig. 6.

In general, the mode profiles of the PLs show a characteristic circular shape owing to the geometry of the core resulting after the tapering process. This arises when the capillaries collapse filling the air gaps among the fibres, and has also been observed during the fabrication of PLs with a smaller number of fibres^19–22^. Regarding the far field intensity-mode profiles, these evolved to become the expected light patterns for the corresponding mode excited in the PL. This is more evident for the lower order modes for both MSPLs, as can be seen upon comparing the LP_01_ and LP_11_ modes with those depicted in Fig. 1.

As seen in the figures, all the estimated LP fibre modes for each of the fabricated MSPLs were obtained upon launching light into the corresponding input fibre. Excitation of the two degenerate modes, corresponding to a same LP mode, was also successfully achieved using the fibres with identical core diameters, but located in symmetrical positions within the arrangement. This is shown only for the field intensity-mode profiles obtained with the 10-fibres device (see Fig. 4), whereas for the 15-fibre device we demonstrate the generation of all the diverse 9 LP modes. In this case, both mode profiles have the exact same intensity distribution but showed a rotation, therefore switching between the spatial location of the minima and the maxima in the intensity patterns.

A point to highlight for the 10-fibres MSPL is the need of a larger core diameter for exciting the LP_02_ compared to the core size used for the LP_31_. Typically, the excitation of the LP_31_ mode would require a smaller core diameter, and inherently a lower propagation constant, than that used to excite the LP_02_ mode. However, the opposite was necessary for our devices, as the LP_02_ mode required a fibre with a smaller core. This is because the microstructured preform comprises dissimilar cladding fibre diameters for the central fibres. This in turn derives in a different distance among the fibre cores affecting the light coupling conditions, and thus the evolution of the modes propagating along the MSPL^26^.

The intensity-mode profiles generated by the MGS-PL exhibit evident mode mixing caused by the superposition of the LP fibre modes and their corresponding degenerate modes within the mode group. Such an effect can be readily observed in the light patterns obtained for groups 2, 3, and is more evident in groups 4 and 5 (see Fig. 6). In spite of this mixing, these PLs might be suitable for applications where mode mixing is acceptable at certain levels, and further reduces the diversity of fibres required for fabrication.

When launching the 980 nm light source into the MSPLs, the registered output intensity distributions were similar to those obtained in the mode profiles with λ = 1550 nm (see Figs 4c and 5c). However, the desired mode profiles were well defined for the higher order modes, whereas the lower order modes showed features typically present in weakly coupled fibre cores, containing independent light spots generated by the remnant light within the core. Such intensity-mode profiles resemble the supermodes necessary to excite the desired LP modes^17,18^. This demonstrates that these devices will show some wavelength dependency, particularly due to the tapering ratio, which was optimized for longer wavelengths. Notice that this effect is reduced when generating the higher order modes, owing to the smaller core diameters of the fibres required to excite these LP modes in MSPLs. Once the tapering process is finished, the smaller fibre core diameters can no longer guide light. Hence, the geometry of the devices must be chosen adequately for proper operation at the desired wavelengths. Nonetheless, these results demonstrate broadband operation of the MSPLs, which is a desirable feature for several applications.

The fibre distributions within the fabricated devices successfully generated the desired LP modes. However, an interesting feature resides in the exploration of different configurations and variations in the fabrication process. Owing to the large amount of possibilities to allocate the fibres and the variation of the core diameters (linked to different propagation constants), this can result in a large number of possible responses. However, most of them will generate output intensity patterns comprised by the mixing of the pure LP modes. To exemplify this phenomenon we fabricated a pair of 10-fibre MSPLs but with slight variations on the fibre configuration used for the devices reported above (Supplementary Figure S1). The results obtained with these PLs can be consulted in the supplementary material.

## Intensity Distribution Mode Profiles of MSPLs

**Figure 7:**
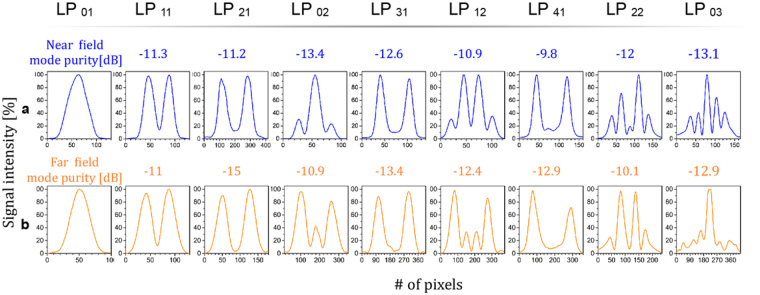
Intensity-distribution mode profiles. (**a**) Near and (**b**) far field plots of the mode profiles for the MSPLs and their respective mode purity.

The mode purities were obtained by measuring the ratio between the minimum and maximum intensities along the spatial distribution of the near and far field mode profiles for the MSPLs^19^. Typical intensity distributions and their corresponding mode purity obtained from both, the near and far field patterns, are shown in Fig. 7. The mode profiles follow closely the spatial intensity distribution expected for each of the excited modes. Furthermore, the evolution of the field during propagation maintains the spatial intensity distribution thus indicating mode preservation, although a change in the intensity peaks is apparent for the LP_02_ and LP_12_ modes.

A remarkable feature of all-fibre photonic lanterns is their minimum losses when compared to other technologies. Hypothetically these devices could be lossless, provided that the tapering process is totally adiabatic^9^. The measured insertion losses for the 10-fibres MSPLs were in the range of 0.1–0.25 dB; similarly, for the 15-fibres devices we measured losses of up to 0.5 dB for the first four mode groups, while for the last mode group these were registered within 1–2 dB. In this case, the higher losses registered for the fifth mode group were mostly due to the low NA of the waveguide structure, resulting in poor light confinement. This could be improved upon using adequate low-index capillaries for the microstructured preforms. It is worth mentioning that the fabrication complexity of PLs increases as the number of modes increases. Furthermore, higher order modes are more susceptible to perturbations and hence to minor imperfections that may arise during the fabrication process.

## Coupling PLs to Optical Fibres

One of the main features of all-fibre photonic lanterns is their ease of integration to optical fibre systems for light/signal transmission. In particular, for SDM applications the goal is to launch the desired LP modes into FMFs. Adequate pairing between the cores of the PLs and the transmission fibres represents a critical factor for a successful alignment, and eventually to excite all the modes supported by the FMFs. Coupling and splicing the lanterns to transmission fibres serve as a typical test to evaluate both, the integration capabilities of the PLs as well as their performance in fibre systems. The capabilities to excite and transmit each of the supported modes of a transmission fibre were evaluated upon splicing the PLs to a FMF.

**Figure 8:**
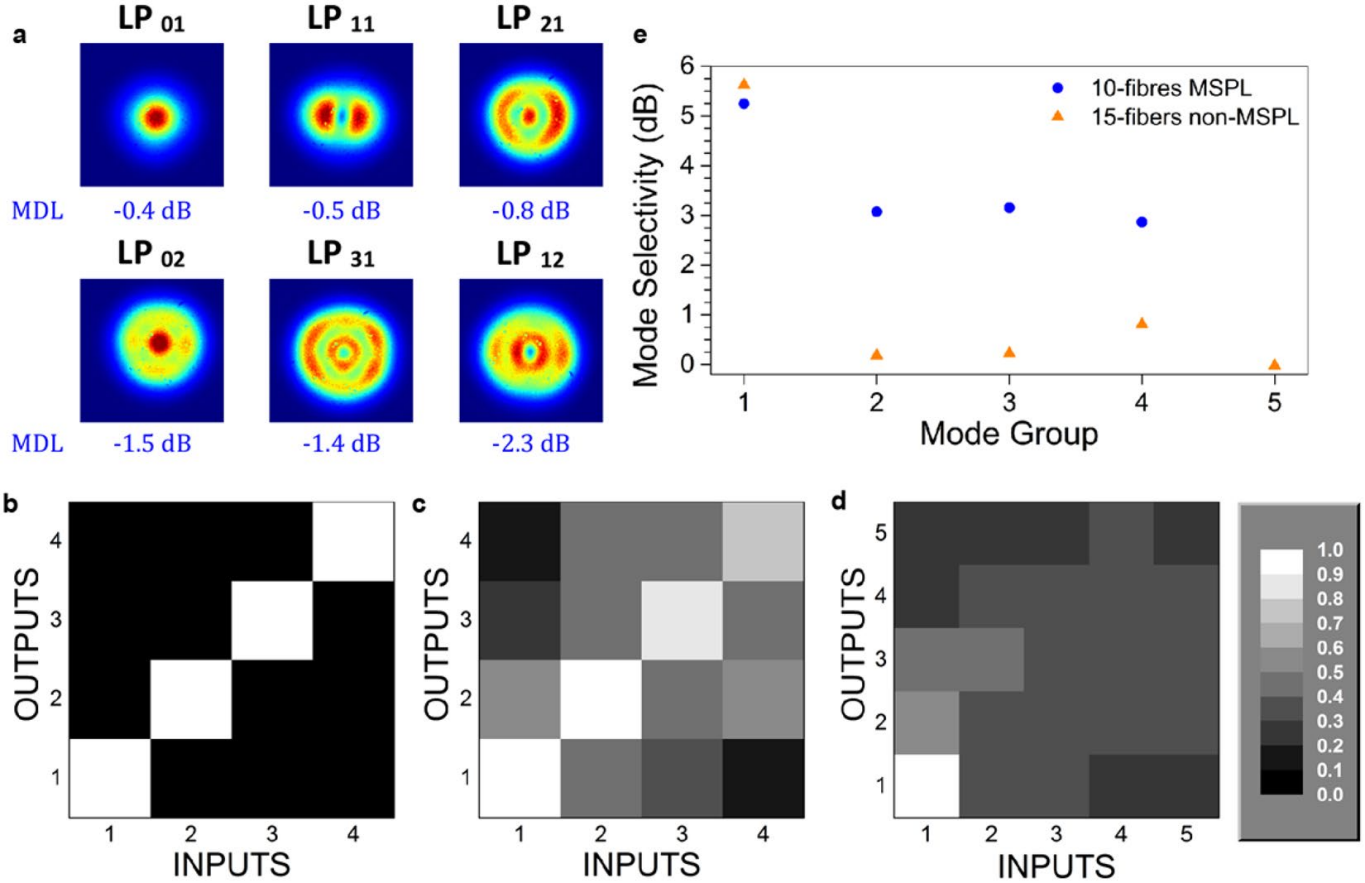
Fibre splice response and mode selectivity analysis of the PLs. (**a**) Near field intensity-mode profiles from a 6-LP modes FMF^27^ spliced to a 10-fibres MSPL, and typical MDL results. Transfer matrix showing the normalized light intensity in mode groups from: (**b**) ideal 10-fibres MSPL, (**c**) experimental 10-fibres MSPL, and (**d**) a 15-fibres PL without modal selectivity. (**e**) Mode selectivity obtained from the comparison between photonic lanterns with modal selectivity (10-fibres MSPLs) and without any modal selectivity (15-fibres non-MSPLs).

The 10-fibres MSPLs were spliced to a 2 m long, low DGD 6-LP FMF with 125 μm outer diameter and a core size of 28 μm in diameter^27^. The characteristic near field intensity-mode profiles registered at the output of the FMF are shown in Fig. 8a; typical coupling losses registered for each mode (MDL) are also included. In general, the lower order modes showed good preservation of the intensity-mode profiles after splicing and propagation through the FMF. In contrast, some mode mixing was observed for the higher order modes. Notice also that the losses increase as the mode group increases. In this case the mismatch of refractive index profiles and NAs between the PL and the FMF yields a difference in the mode overlap and thus leads to mode coupling^28^. This increases the MDL for the higher order modes aside from the intrinsic MDL of the fibers^28,29^. The use of a PL with an adequate core geometry and graded-index profile will improve the MDL^30^.

Due to the low NA of the 15-fibres PLs, the final diameter obtained for this device was limited to a core size of 35 μm prior to mode leakage. Direct splicing to a 9-LP FMF (30 μm core diameter) was therefore not possible due to the large core mismatch and NA difference^31^. To circumvent this limitation, an intermediate standard MMF (50 μm core diameter and gradual index profile) was used for core matching purposes. The MMF was tapered to reduce the core diameter and match the size of the 9-LP FMF core, and also served to restrict the propagation of unnecessary modes. In spite of this procedure, the intensity-mode profiles at the output of the FMF (not shown) revealed mode mixing, and the measured MDL values were close to 5 dB.

Characterization of the PLs as MUX/DEMUX devices was performed upon splicing pairs of lanterns onto opposite ends of the FMFs. Light from a swept wavelength interferometer (SWI) with spatial diversity was then launched into every input fibre of the MUX, and the output power at each of the DEMUX fibres was measured. This arrangement allows obtaining the transfer matrix along the entire C- and L-bands, commonly used for optical fibre communications^32^. The transfer matrix plots the normalized intensities at the inputs and outputs of the PLs used for MUX/DEMUX purposes, indicating how the light sent at each input fibre from the MUX lantern is transmitted and coupled to the rest of the LP or mode groups. An important feature of this graphic representation is the capability to provide information about crosstalk among the spatial channels, as well as information regarding mode mixing. This in turn allows to determine the mode selectivity of the system, defined as the ratio of the power in a mode group between the total power contained in all the mode groups. Ideally, a system with absolute modal selectivity should present zero mode mixing and negligible crosstalk. Thus, for an ideal MSPLs and FMF showing only mode mixing within the mode groups, the total power launched at any single fibre will be equally distributed only among the corresponding fibres to the same mode group (see Fig. 8b). In contrast, for a system with no modal selectivity at all, the light launched at any input fibre will spread evenly among all of the output fibres.

Two cases were examined in order to compare different scenarios: a system using mode-selective lanterns (10-fibres MSPLs), and the other using PLs without any modal selectivity (15-fibres non-MSPLs). The PLs without mode selectivity were fabricated using identical fibres (13 μm core diameter), following the procedure described earlier. Using the same fibres for the PLs yielded devices whose output light patterns are an orthogonal combination of all the supported modes. The transfer matrices obtained for both cases are shown in Fig. 8c and d. As evidenced in the figure, the light is mostly contained in the central diagonal for the mode selective case, whereas the transfer matrix without modal selectivity shows that light spreads throughout all of the supported mode groups. Upon evaluating the ratio of the measured power in the desired mode to the power measured for all of the remaining modes in a mode group, we can estimate the mode selectivity of the MUX/DEMUX-FMF system. A comparison of this parameter for both of the fabricated devices is shown in Fig. 8e. For the first mode group (LP_01_), both devices show similar performance for mode isolation. However, for the remaining mode groups the mode-selective system shows that twice the power is preserved within a group mode when compared to the non-mode-selective system. This performance can be further improved upon employing PLs with a core having a better match with the FMFs^30^. For higher order mode groups the MSPL system clearly shows a better performance for mode selectivity. Hence, for a system with non-mode selective MUX/DEMUX devices, mode crosstalk will occur during propagation, particularly for higher order modes.

## Conclusions

We have demonstrated the fabrication of low-loss all-fibre photonic lanterns based on the use of microstructured preforms. The preforms are key elements that allow positioning the launching fibres in correct locations to generate a desired mode profile. Furthermore, this approach enables for a simpler and repeatable fabrication process suitable for obtaining devices that require a large number of fibres. Upon the correct design of the fibres and their position within the preform, we fabricated 10- and 15-fibre photonic lanterns with modal selectivity, capable to generate up to 9 LP modes with high purity. The main features of these devices, such as mode dependent loss and wavelength dependency were also analysed, showing promising results for SDM applications. We further explored the use of these PLs as MUX/DEMUX devices spliced to FMFs and evaluated the performance of the arrangement through the transfer matrix. Our results show that proper mode selectivity is sustained after transmission through FMF, confirming that the PLs are correctly designed as mode selective devices. Hence, all-fibre PLs hold as attractive candidates for developing the next generation of multiplexers required in SDM systems.

## Methods

Assembly of the photonic lanterns was performed by manually placing the required number of fibres inside the microstructured preforms in the correct positions, according the desired number of modes at the output of the PL. The fibres were inserted after removing a section of the polymer coating. An optical microscope with a long work distance objective was used to achieve the correct placement of each fibre within the structure. The whole structure was then tapered using a CO_2_ laser glass processing station (LZM-100, AFL Fujikura). The operational principle of the station is based on the heat-and-pull method. The devices were then cleaved at the taper waist once the final diameter was reached.

The microstructured capillaries and optical fibres used for the construction of the photonic lanterns were fabricated at CREOL The College of Optics & Photonics, at the University of Central Florida, USA.

The output mode profiles of the photonic lanterns were obtained with an IR camera, XEVA-1.7-320 from Xenics. The intensity distributions were analysed with the Xeneth 2.6 software from Xenics, obtaining the signal intensities per pixel. Mode purity values were obtained by normalizing each mode profile with respect to its maximum intensity value. The ratio between the minimum and maximum normalized intensities along the spatial distribution^19^ was obtained from:

$${\rm{Mode}}\,{\rm{Purity}}=10\,{\mathrm{log}}_{10}({I}_{MAX}/{I}_{MIN})$$

Transfer matrices were measured using a swept wavelength interferometer (SWI)^32^ with spatial diversity. Light was launched into each input fibre of the MUX while measuring the output power for each output of the DEMUX photonic lantern. The transfer matrix plots were obtained using the normalized intensities at the inputs and outputs of the PLs.

## Electronic supplementary material

Supplementary figure S1

## References

[1] Essiambre RJ, Kramer G, Winzer PJ, Foschini GJ, Goebel B (2010). Capacity Limits of Optical Fibre Networks. J. Lightwave Technol..

[2] Richardson DJ, Fini JM, Nelson LE (2013). Space-division multiplexing in optical fibres. Nat. Photonics.

[3] Sillard P (2015). Next-Generation Fibres for Space-Division-Multiplexed Transmissions. J. Lightwave Technol..

[4] Van Uden RGH (2014). Ultra-high-density spatial division multiplexing with a few-mode multicore fibre. Nat. Photon..

[5] Mizuno T, Takara H, Sano A, Miyamoto Y (2016). Dense Space-Division Multiplexed Transmission Systems Using Multi-Core and Multi-Mode Fibre. J. Lightwave Technol..

[6] Ryf R (2012). Mode-Division Multiplexing Over 96 km of Few-Mode Fibre Using Coherent 6 × 6 MIMO Processing. J. Lightwave Technol..

[7] van Weerdenburg J (2015). 10 Spatial mode transmission using low differential mode delay 6-LP fibre using all-fibre photonic lanterns. Opt. Express.

[8] Birks TA, Gris-Sánchez I, Yerolatsitis S, Leon-Saval SG, Thomson RR (2015). The photonic lantern. Adv. Opt. Photon..

[9] Leon-Saval SG, Fontaine NK, Amezcua-Correa R (2017). Photonic lantern as mode multiplexer for multimode optical communications. Opt. Fibre Technol..

[10] Igarashi K, Souma D, Takeshima K, Tsuritani T (2015). Selective mode multiplexer based on phase plates and Mach-Zehnder interferometer with image inversion function. Opt. Express.

[11] Labroille, G., Jian, P., Barré, N., Denolle, B. & Morizur, J. F. Mode Selective 10-Mode Multiplexer based on Multi-Plane Light Conversion. In *Optical Fiber Communications Conference and Exhibition* (*OFC*), *Anaheim*, *California United States*, Th3E.5, 10.1364/OFC.2016.Th3E.5 (2016).

[12] Melati D, Alippi A, Annoni A, Peserico N, Melloni A (2017). Integrated all-optical MIMO demultiplexer for mode- and wavelength-division-multiplexed transmission. Opt. Lett..

[13] Xia C (2015). Time-division-multiplexed few-mode passive optical network. Opt. Express.

[14] Fontaine, N. K. *et al*. 30 × 30 MIMO Transmission over 15 Spatial Modes. In *Optical Fiber Communications Conference and Exhibition* (*OFC*), *Los Angeles*, *California United States*, Th5C.1, 10.1364/OFC.2015.Th5C.1 (2015).

[15] Van Newkirk A (2015). Bending sensor combining multicore fibre with a mode-selective photonic lantern. Opt. Lett..

[16] Wittek S (2016). Mode-selective amplification in a large mode area Yb-doped fibre using a photonic lantern. Opt. Lett..

[17] Ryf R, Fontaine NK, Essiambre RJ (2012). Spot-Based Mode Couplers for Mode-Multiplexed Transmission in Few-Mode Fibre. IEEE Photon. Technol. Lett..

[18] Fontaine NK, Ryf R, Bland-Hawthorn J, Leon-Saval SG (2012). Geometric requirements for photonic lanterns in space division multiplexing. Opt. Express.

[19] Yerolatsitis S, Gris-Sánchez I, Birks TA (2014). Adiabatically-tapered fibre mode multiplexers. Opt. Express.

[20] Leon-Saval SG (2014). Mode-selective photonic lanterns for space-division multiplexing. Opt. Express.

[21] Velazquez-Benitez AM (2015). Six mode selective fibre optic spatial multiplexer. Opt. Lett..

[22] Huang B (2015). All-fibre mode-group-selective photonic lantern using graded-index multimode fibres. Opt. Express.

[23] Velázquez-Benítez, A. M. *et al*. Scaling the fabrication of higher order photonic lanterns using microstructured preforms. In *European Conference on Optical Communication* (*ECOC*), *Valencia*, *Spain*, Tu.3.3.2, 10.1109/ECOC.2015.7341939 (2015).

[24] Yerolatsitis, S., Harrington, K., Thomson, R. & Birks, T. A. Mode-selective Photonic Lanterns from Multicore Fibres. In *Optical Fiber Communications Conference and Exhibition* (*OFC*), *Los Angeles*, *California United States*, Tu3J.6, 10.1364/OFC.2017.Tu3J.6 (2017).

[25] Eznaveh ZS (2017). All-fiber few-mode multicore photonic lantern mode multiplexer. Opt. Express.

[26] Leon-Saval SG, Argyros A, Bland-Hawthorn J (2010). Photonic lanterns: a study of light propagation in multimode to single-mode converters. Optics Express.

[27] Sillard, P. *et al*. Low-DMGD 6-LP-Mode Fibre. *In Optical Fiber Communications Conference and Exhibition* (*OFC*), M3F.2, 10.1364/OFC.2014.M3F.2 (2014).

[28] Warm S, Petermann K (2013). Splice loss requirements in multi-mode fiber mode-division-multiplex transmission links. Opt. Express.

[29] Ho KP, Kahn JM (2011). Mode-dependent loss and gain: statistics and effect on mode-division multiplexing. Opt. Express.

[30] Zacarias, J. C. A., *et al*. Mode Selective Photonic Lantern with Graded Index Core In *Optical Fiber Communications Conference and Exhibition* (*OFC*), *Los Angeles*, *California United States*, M4D.5, 10.1364/OFC.2018.M4D.5 (2018).

[31] Sillard P (2016). Low-Differential-Mode-Group-Delay 9-LP-Mode Fibre. J. Lightwave Technol..

[32] Fontaine, N. K. *et al*. Characterization of Space-Division Multiplexing Systems using a Swept-Wavelength Interferometer In Optical Fiber Communication Conference and Exposition and the National Fiber Optic Engineers Conference (OFC/NFOEC) Anaheim, California United States, OW1K.2, 10.1364/OFC.2013.OW1K.2 (2013).

