# Supplementary material for: Scaling photonic lanterns for space-division multiplexing
Source: Sci Rep. 2018 Jun 11;8:8897. doi: 10.1038/s41598-018-27072-2 (PMC5995907; doi:10.1038/s41598-018-27072-2)
Supplement: Supplementary file 1 — Supplementary figure S1 [file 41598_2018_27072_MOESM1_ESM.pdf]

Scaling photonic lanterns for space-division multiplexing

Amado M. Velázquez-Benítez[1,2,3,7,*], J. Enrique Antonio-López[1], Juan C. Alvarado-Zacarías[1], Nicolas K. Fontaine[2], Roland Ryf[2], Haoshuo Chen[2], Juan Hernández-Cordero[3], Pierre Sillard[4], Chigo Okonkwo[5], Sergio G. Leon-Saval[6], and Rodrigo Amezcua-Correa[1,*]

[1]CREOL, The College of Optics & Photonics, the University of Central Florida, Orlando, Florida 32816-2700, USA

[2]Bell Laboratories/Alcatel-Lucent, 791 Holmdel Rd., Holmdel, New Jersey 07733, USA

[3]Instituto de Investigaciones en Materiales, UNAM, Cd Universitaria, Ciudad de México, 04510, México

[4]Prysmian Group, Parc des Industries Artois Flandres, 644 boulevard Est, Billy Berclau, 62092 Haisnes Cedex, France

[5]Institute for Photonic Integration, Flux, Groene Loper 5, 5612 AE, Eindhoven, The Netherlands

[6]Institute of Photonics and Optical Science, School of Physics, The University of Sydney, New South Wales, 2006, Australia

7Current address: Instituto de Ciencias Aplicadas y Tecnología, UNAM, Cd. Universitaria, Mexico City, 04510, México

*E-mail: amadovelb@comunidad.unam.mx r.amezcua@creol.ucf.edu

Supplementary figure S1

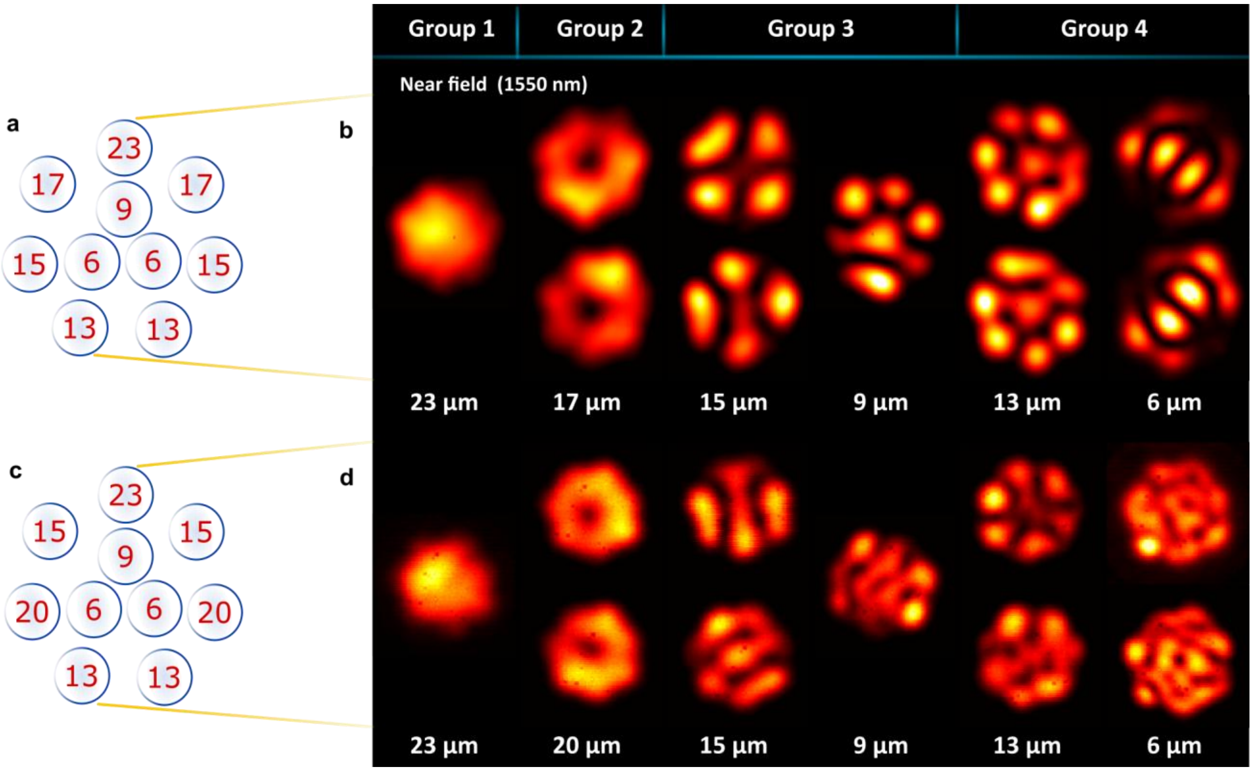

Supplementary figure S1 | Different configuration of fibres for 10-fibre PLs. Ten fibre PLs fabricated using similar fibre core diameters located, at similar positions, and using the same fabrication parameters (e.g., tapering ratio, length, etc.) as those used for the ten fibre shown in Figure 3a. PL fabricated with different fibre diameters positions reducing the sizes of two of the fibre cores (one changed from 20 to 17 µm and the other from 17 to 15 µm): (**a**) fibre core distributions and (**b**) near field mode profiles. PL fabricated using different fibre positions, with one fibre core reduced (from 17 to 15 µm) and two fibres swapped from their original positions: (**c**) fibre core distributions and (**d**) near field mode profiles.
